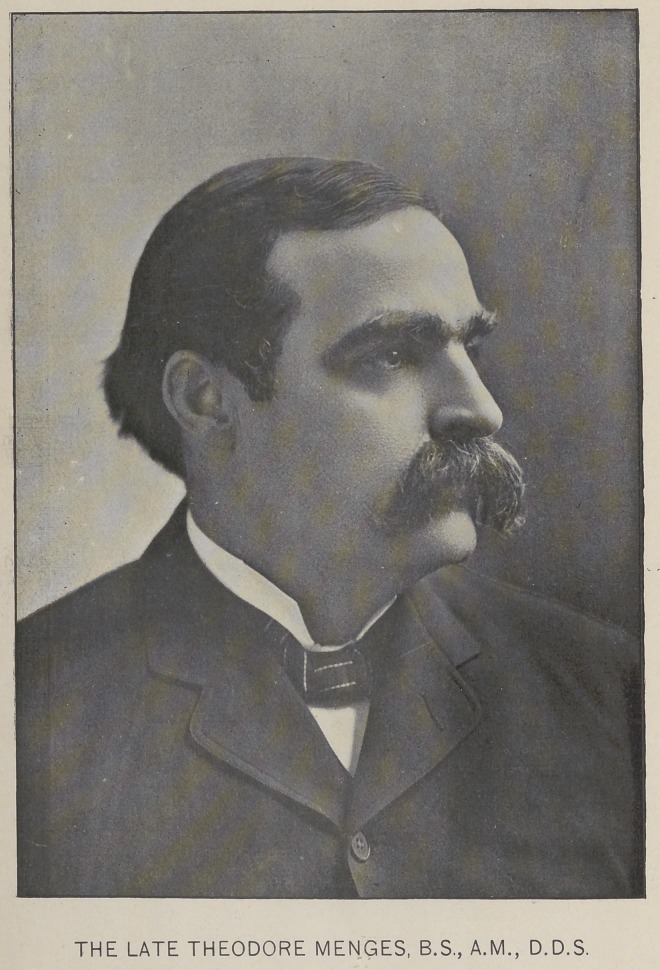# Theodore Menges, B.S., A.M., D.D.S.

**Published:** 1900-06-15

**Authors:** G. V. Black


### THEODORE MENGES, B.S, A.M., D.D.S.

Theodore Menges died June 1, 1900, in Chicago, after an illness of a little less than one week. He was born near Bristol, Ind., May 2, 1854. His early boyhood was spent on his father's farm at the ordinary employments, and in the country school. In the school he excelled, and at the early age of sixteen he became the teacher, which place he held for five years.

During these years he spent a part of his summers at the North Indiana Normal School and earned the B. S. degree. Subsequently he received the degree of A. M. After these first five years of school work he took charge of a larger school at Bloomfield, Ind., and soon afterward organized a normal school at the same place, which was successful. This occupied his time for seven years. In 1879 he was married to Miss Alice Brown, one of the teachers in his school. During the latter part of this school work he was diligently studying law, and was admitted to the bar at the close of his school work and entered into practice at Bloomfield. About the time he gave up teaching he took one course in the Jefferson Medical College at Louisville, Ky., studying the fundamental branches and returned at once to the law. He remained at Bloomfield but two years as a lawyer. He went West, first to Kimball, Nebraska, and later to Cheyenne, Wyoming, continuing in the law and engaging in real estate and mining speculations, for which he developed a taste and much ability. Within a few years he had amassed what seemed to be a large fortune but with the death of the boom in Western lands and mining property ruin seemed inevitable, but by skillful management he saved something from the wreck and after winding up the businsss came to Chicago in 1891. Here both he and Mrs. Menges entered the American College of Dental Surgery, from which each received the degree of Doctor of Dental Surgery in due course. During his pupilage Dr. Menges had acquired a taste for dental school affairs and upon his graduation assumed the supervision of the school as its secretary. His wide acquaintance with educational matters, his untiring energy, combined with good judgment, enabled him to quickly infuse new life into the school. Its classes grew rapidly in numbers, the quality of the instruction was greatly improved, the equipment increased and made better in still greater ratio than the increase of the classes, and the school put upon a strictly educational basis that merited confidence in its future.

In 1896 the American College of Dental Surgery was consolidated with Northwestern University Dental School, their classes combined, and Dr. Menges was retained by the University as its representative. The school prospered and became rapidly the largest in the numbers of its students in the world and one among the best. He has built up a library, museum and reading room, which, for a dental school, may be justly regarded as great, and the whole equipment and management was being rapidly improved when he was suddenly cut off while yet in middle life and in the full strength of his manhood.

Dr. Menges had entered rapidly into active and intimate relationship with the dental profession. He was an active member of several of the local dental societies in Chicago, of the Illinois State Dental Society, the National School of Dental Technics, the Dental Protective Association, the National Dental Association and the National Association of Dental Faculties. It was perhaps in the National Association of Dental Faculties that he had made the greatest impression upon the dental world. He was the Chairman of its Executive Committee, the attitude and action of which had more influence in shaping and moulding educational matters in dentistry than any other organization in the world. In this work he was active, skillful and aggressive, always standing for higher ideals in educational work. In the period of nine years from the time of beginning the study of dentistry he had become a national figure in educational matters.

At the time he was stricken down he was studying the subject of dental education with much zeal with the view eventually of bringing about a better understanding of the subject among teachers, the profession at large and in the several schools. In this work his ideas and intentions were broad and liberal in method toward all who might desire decisive improvement upon the present. Really, he seemed to be just getting started in the work, especially in arranging the matter in his mind with the promise of writing effectively. He was naturally impetuous in everything in which he was keenly interested, and fully realized this as a personal fault, which he was laboring to reduce to the minimum consistent with his great personal activity. The great ambition of his life had come to be to effect a radical improvement in dental education, not especially in his own school, but in all of the schools of the country. He had lately acquired a profound conviction that the weaker schools must be improved as the first step which would enable the stronger schools to move forward safely and efficiently. In comparison with his desires in these directions personal financial considerations were of little consequence. While he laid stress upon financial matters in school work, it was always as a means to the nobler end. Neither did he hesitate to use his private funds without hope of return when such use would seem to effect the good he sought to accomplish. Higher dental education has lost a powerful advocate.

In his private life Dr. Menges was a simple, unostentatious person but of commanding presence. In conversation he was earnest, eager, bright and often very attractive. This was especially true of him in his calmer moments when, with a few friends, he laid aside his business activities. It was then that his true, genial, warm-hearted character shown out the brightest and made his companionship a pleasure.

He leaves no family except his wife, who has constantly been with him but assisting him in his school work, and who has developed an interest and a zeal closely akin to his own. G. V. BLACK.

## Figures and Tables

**Figure f1:**